# Harnessing Li‐Ion Binding Energy for Solvation‐Guided Electrolyte Additive Design and Robust Solid Electrolyte Interphase Reinforcement

**DOI:** 10.1002/advs.77072

**Published:** 2026-08-10

**Authors:** Jooeun Byun, Ho Yeon Jang, Myung‐Jun Kwak, Chae Rim Lee, Chihyun Hwang, Seoin Back, Hyun‐seung Kim

**Affiliations:** ^1^ Department of Advanced Materials Science and Engineering Sungkyunkwan University Suwon Republic of Korea; ^2^ Department of Chemical and Biomolecular Engineering Institute of Emergent Materials Sogang University Seoul Republic of Korea; ^3^ Advanced Batteries Research Center Korea Electronics Technology Institute Seongnam Republic of Korea; ^4^ KU‐KIST Graduate School of Converging Science and Technology Korea University Seoul Republic of Korea; ^5^ Department of Integrated Energy Engineering Korea University Seoul Republic of Korea; ^6^ Institute for Multiscale Matter and Systems (IMMS) Ewha Womans University Seoul Republic of Korea; ^7^ Department of Energy Sungkyunkwan University Suwon Republic of Korea; ^8^ Department of Future Energy Engineering Sungkyunkwan University Suwon Republic of Korea; ^9^ Department of Battery Science and Engineering Sungkyunkwan University Suwon Republic of Korea

**Keywords:** electrolyte additive, lithium‐ion, lithium‐ion batteries, solid electrolyte interphase, solvation

## Abstract

The rational design of electrolyte systems is essential for stabilizing the electrode−electrolyte interface to enhance the electrochemical performance of lithium‐ion batteries (LIBs). Although conventional strategies focus on the introduction of specific functional moieties, the correlation between the molecular structure of an electrolyte additive and the extent of effective localized enrichment at the interface remains poorly understood. Herein, we propose a solvation‐guided additive design strategy in which the Li‐ion binding energy of the additive serves as a factor for effective solid electrolyte interphase (SEI) reinforcement. This hypothesis is validated by employing a σ‐bonding‐insulation‐based model system, which contains allyl methyl sulfone (AMS), allyl methyl carbonate (AMC), and allyl methyl ether (AME) as electrolyte additives with varying Li‐ion binding affinities. Computational simulations and spectroscopic analyses confirmed that AMS, which has the highest binding energy, preferentially participates in the Li‐ion solvation shell. This affinity, which facilitates the spontaneous migration of the additive toward the negative electrode during the SEI formation step, results in the development of a robust SEI layer concentrated at the negative electrode. These findings highlight the importance of interactions between Li‐ions and additives in interface engineering and provide a systematic framework for the development of high‐performance electrolyte additives.

## Graphical abstract

A solvation‐guided design strategy for electrolyte additives is established, identifying Li‐ion binding energy as a crucial determinant for effective interface reinforcement.

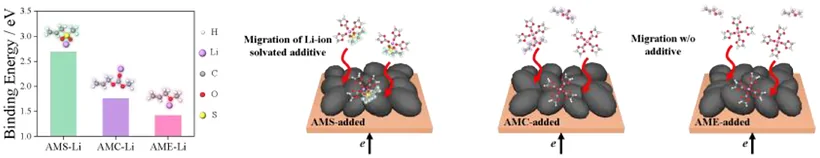

## 1 Introduction

Electrolyte additives designed to stabilize the electrode interface are widely used to enhance the electrochemical performance of secondary batteries [1, 2, 3, 4, 5, 6, 7, 8, 9, 10, 11, 12, 13, 14, 15, 16, 17]. Specifically, the stability of the interface between the electrolyte and the surface of the negative electrode is essential because the additional solid electrolyte interphase (SEI) film that forms after the initial SEI formation cycle enables the consumption of Li ions and electrons in the electrolyte within the confined volume of lithium secondary batteries [18, 19, 20, 21, 22, 23, 24, 25, 26, 27, 28]. In this context, the introduction of the well‐known vinylene carbonate additive serves to stabilize the surface of the negative electrode [11, 29, 30, 31, 32, 33], which, in the case of a graphite electrode, is known to ensure highly stable cycling performance.

In general, the SEI film is modulated by introducing functional moieties, which form a typical compositional SEI film [13, 18, 23, 24, 25, 26, 34, 35, 36]. The incorporation of fluorine‐ [37, 38], sulfur‐ [12, 39, 40, 41], and nitrogen‐ [3, 6, 36, 42, 43] based functional groups into SEI films is widely known to stabilize the interface of the negative electrode; hence, the introduction of such species by way of an electrolyte additive is a typical approach for designing an SEI‐forming additive. Yet, the introduction of functional species into the electrolyte (and thus into the SEI film) generally does not necessarily improve the cycling performance and stability of secondary batteries [2, 44, 45]. Instead of introducing specific functional groups, we propose that the Li‐ion binding energy of the electrolyte additive is a crucial determinant of the extent of reinforcement of the SEI film that forms on the surface of the negative electrode. Sufficient coverage of the graphite electrode surface by the electrolyte additive is crucial for the formation of a functional passivation film. Because the applied external current is divided among graphite lithiation, SEI formation from the solvent, and SEI formation by functional additives, a high concentration of the SEI‐forming additive near the electrode surface is a prerequisite for the effective formation of the SEI film on the graphite electrode by the functional additive.

Typically, the SEI‐forming additive is a highly polar molecule derived from functional moieties [4, 36, 46, 47, 48, 49, 50, 51, 52]; therefore, the SEI‐forming additive should ideally interact with Li ions after the SEI‐forming additive is dissolved in the electrolyte. This is crucial because the migration of Li ions toward the surface of the negative electrode must occur during the formation step to maintain the charge balance of the electrochemical cell. Correspondingly, the additive to which the Li ions are attached forges ahead during the formation period compared to an additive with a lower affinity for Li‐ions. This spontaneous mechanism could be an important contributor to the effectiveness of SEI film‐forming additives. Although previous studies have suggested that the interaction between Li‐ions and solvent molecules can influence SEI formation at thermodynamic perspective [53], the actual SEI formation process occurs under continuously changing negative electrode potentials. Furthermore, in contrast to solvent molecules, electrolyte additives are incorporated at relatively low concentrations in the electrolyte. Therefore, for an additive to effectively participate in SEI formation, its transport to the negative electrode surface is expected to play an important role. However, this aspect has not been systematically investigated in previous studies. To address this knowledge gap, we designed a comparative model system in which the SEI‐forming moiety was kept identical while only the Li‐ion binding energy was varied. This approach enabled us to isolate the effect of Li‐ion binding energy from differences in decomposition chemistry and directly evaluate its influence on additive behavior during SEI formation. Through this comparative study, we aimed to demonstrate that Li‐ion binding energy should be considered as an important design parameter for developing effective SEI‐forming electrolyte additives.

This motivated our study, which was designed to verify this hypothesis and was based on a comparison of σ‐bonding‐insulating molecules. The major SEI‐film‐forming moiety in the applied model compound was unified as an allyl moiety, which was insulated with a single aliphatic chain with Li‐ion‐interacting moieties. Application of this model compound enabled us to conduct a precise comparative study to identify an advanced determinant capable of stabilizing the negative electrode–electrolyte interface using functional additives.

Focusing on the selected group of model additives, we used computational chemistry and spectroscopic analyses to investigate whether the introduction of these additives resulted in the expected effects within the local solvation structure. Furthermore, we examined whether such local solvation structures influenced the effective formation of a SEI layer. Based on these analyses, we confirmed that additives with stronger binding affinities for Li ions contribute to the formation of a more effective SEI layer associated with the negative electrode.

The impact of the initially reinforced SEI layer on the cell lifetime was evaluated by conducting electrochemical testing in 1.0 A h‐class cells incorporating the corresponding additives. During this evaluation, the additives were compared based on their molar concentrations. This enabled an assessment of the additive effects under equivalent conditions. For instance, the concentrations of species generating decomposition currents in actual electrochemical reactions were equivalent. Finally, X‐ray microscopy was employed to quantitatively analyze the influence of the additive design strategy on the pore‐clogging phenomena within the electrode arising from the suppression of continuous side reactions. This experimental design made it possible to systematically elucidate the effects of the binding strength between the Li ions and the additive on the cell performance.

## 2 Results and Discussion

Figure 1 depicts the structure of the additive model, which was designed based on the σ‐bonding‐insulation model, and the change in the microsolvation structure of the electrolyte induced by the binding affinity (quantified by the binding energy) for Li ions. Figure 1a shows the charge density of the models of the allyl methyl sulfone (AMS), allyl methyl carbonate (AMC), and allyl methyl ether (AME) molecules. The sequence of the charge densities of the solvation moieties, sulfone, carbonate, and ether, is well correlated with the Li‐ion binding energies shown in Figure 1b, implying that strong solvation is expected with a highly polarized solvation moiety. To further validate this in the electrolyte system, binding energies within the primary solvation shell were calculated (Figure S1). The results confirm that AMS possesses the strongest thermodynamic driving force to displace bulk solvents (EC or EMC) and dominate the solvation structure. Furthermore, to account for the ion‐pairing effects prevalent in higher‐concentration electrolytes, we additionally evaluated the binding affinities within a contact ion pair (CIP) configuration ([Li(PF_6_)(EC)(EMC)]). As illustrated in Figure S2, although the coordination of the PF_6_^−^ anion uniformly reduces the binding energies, the relative trend (AMS > AMC > AME) is preserved. This confirms that the superior solvation preference of AMS remains robust across varying coordination environments.

**FIGURE 1:**
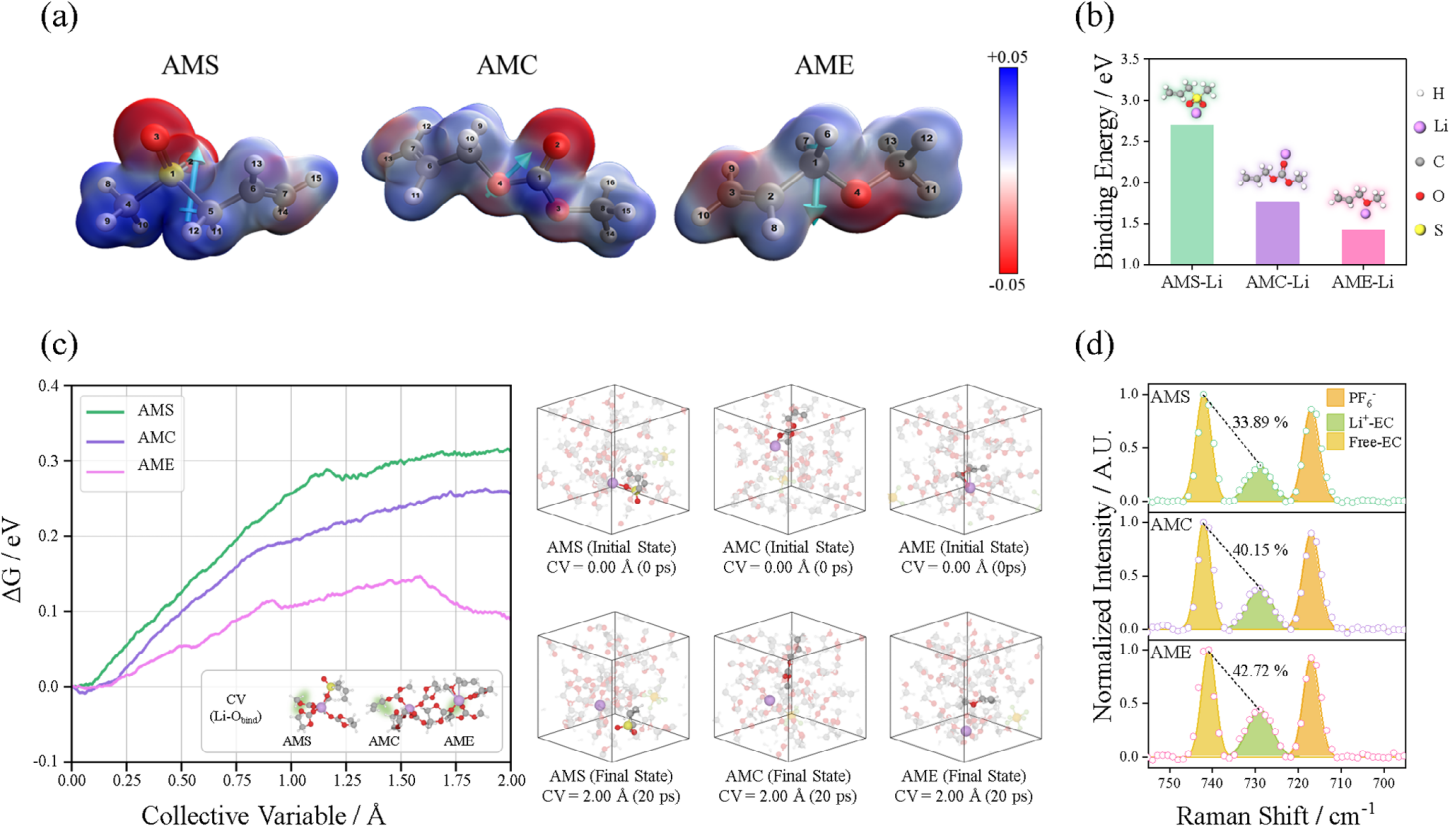
(a) Electrostatic potential (ESP) maps for the optimized additive molecules [allyl methyl sulfone (AMS), allyl methyl carbonate (AMC), and allyl methyl ether (AME)], plotted on an iso‐surface of 0.01 e Å^−3^; (b) comparison of Li‐ion binding energy; (c) free energy profiles (∆G) for the detachment of each additive from the initial [Li(EC)_2_(EMC)(additive)]^+^ solvation structure, calculated using slow‐growth ab initio molecular dynamics (AIMD) simulations. The collective variable (CV) is defined as the Li‐O_bind_ distance (inset, highlighted in green). The panels on the right display snapshots showing the initial state (CV = 0.0 Å at 0 ps) and the fully detached final state (CV = 2.0 Å at 20 ps). The Li^+^‐additive complex is highlighted to clearly visualize the detachment process. The purple, gray, red, white, and yellow spheres correspond to Li, C, O, H, and S atoms, respectively, and (d) Raman spectra of the electrolytes with the AMS, AMC, and AME additives, respectively.

The most important finding of the σ‐bonding‐insulation‐based model system is that the lowest unoccupied molecular orbital (LUMO) energy levels of Li‐ion‐attached AMS, AMC, and AME are similar, indicating that a thermodynamic preference for electrochemical reduction would not be expected from this tailored model system design (Figure S3). Consistent with the similar LUMO energy levels, the linear sweep voltammetry (LSV) profiles obtained using Li/Cu cells (Figure S4) exhibited highly similar reduction onset potentials at around 1.5 V (vs. Li/Li^+^) among AMS, AMC, and AME. In addition, the cathodic peak positions and current responses showed no significant differences among the three electrolytes, indicating comparable electrochemical reduction behavior. These observations supportthe DFT results. The simulations were randomly initialized by including two different pre‐optimized solvation structures (i.e., [Li(EC)_2_(EMC)(additive)]^+^ or [Li(EC)(EMC)_2_(additive)]^+^) with the remaining free molecules and anions (labeled “Random Structure” in Figures S5 and S6) using the Packmol package [54]. All ab initio molecular dynamics (AIMD) simulations were performed under the NVT ensemble at 330 K using a Nosé–Hoover thermostat to reflect the battery operating conditions [2, 55]. The simulation procedure began with a 20 ps pre‐optimization starting from the randomly initialized structures (Figures S5 and S6). Throughout this stage, the central Li–(O)_4_ cluster was fixed to equilibrate the surrounding solvent environment. Subsequently, all atomic constraints were released, and a 30 ps AI‐MD simulation was performed. The first 10 ps of this trajectory was treated as the final equilibration period, and the subsequent 20 ps was designated as the production phase for data analysis. The time step for all AI MD simulations was 1fs.

The dynamic solvation behaviors of the additive molecules were investigated by performing AIMD simulations (Figures S7 and S8). Only the AME additive detached from the initial [Li(EC)_2_(EMC)(additive)]^+^ solvation structure. Although this spontaneous detachment highlighted that AME is weakly bound, a more systematic comparison was required to evaluate the relative detachment tendencies of all three additives. The detachment free energies were quantified by performing slow‐growth AIMD simulations starting from the initial [Li(EC)_2_(EMC)(additive)]^+^ solvation structures. (Figure 1c). The collective variable (CV) was defined as the distance between the Li^+^ and the binding oxygen atom of the additive (Li‐O_bind_). As shown in Figure 1c, the free energy profiles for detachment indicated that the energy required for dissociation increased in the order AME < AMC < AMS. This trend, obtained from the dynamic detachment process, agrees with the static binding energy results (Figure 1b), confirming that AMS has the highest binding energy among the additives. This consistent detachment trend was verified for the alternative [Li(EC)(EMC)_2_(additive)]^+^ initial solvation structure, which further supported the strong binding of AMS (Note S1; Figures S9 and S10). Although our models describe Li^+^ solvation in the bulk regions rather than explicit solid–liquid interfaces, recent studies indicate that SEI nucleation often begins with the reductive decomposition of solvated complexes in the electric double layer (EDL) [2, 55]. To link these solvation preferences to SEI formation, we calculated the one‐electron reductive energies of isolated additives and their corresponding solvation structures (Figure S11). The results show that coordination with Li^+^ significantly lowers the reductive energy across all additives. This indicates that a strong Li^+^ binding affinity not only promotes the inclusion of additives into the primary solvation shell but also facilitates their subsequent reduction, thereby contributing to early‐stage SEI formation.

Figure S12 shows the concentration profiles of the Li‐ion‐attached and ‐detached species of the AMS, AMC, and AME molecules. The concentration profile shows that AMS has the greatest tendency to attach itself to Li ions, followed by the AMC and AME additives. The AIMD results were verified using Raman spectroscopy (Figure 1d). The ratio of ethylene carbonate (EC) molecules attached to Li ions relative to free EC molecules was calculated because the replacement of EC with the model additive induces an increase in the concentration of free EC in the electrolyte. As expected from the AIMD simulation, the relative Li‐ion‐bound EC concentration was in the sequence AME, AMC, and AMS as the electrolyte additives. Thus, the results for the solvation microstructure from the AIMD simulations were in full agreement with those observed by Raman spectroscopy. The concentration‐dependent ionic conductivity was measured to compare the solvation ability (Figure S13). The substitution of EC or EMC molecules in the solvation cluster by the electrolyte additive generally increases the volume of solvated Li ions, implying that the decrease in ionic conductivity is significant with the introduction of the additive. The decrease in ionic conductivity from 1.0 to 3.0 wt.% of the additives is 0.25, 0.19, and 0.17 mS cm^−1^ for AMS, AMC, and AME, respectively. Additives with strong Li‐ion affinity can participate in the Li‐ion solvation structure by replacing EC and EMC molecules within the primary solvation shell. Because such solvent substitution is expected to influence ion transport properties, the ionic conductivity was evaluated as a function of additive concentration to indirectly assess the extent of additive incorporation into the solvation structure. As the additive concentration increased, the decrease in ionic conductivity was the most pronounced for AMS, followed by AMC and AME. This trend is consistent with the predicted order of solvent substitution derived from the binding energy calculations and AIMD simulations, suggesting that AMS exhibits the highest degree of participation in the Li‐ion solvation structure.

Because the initial adsorption of additive on the graphite surface can affect the SEI‐formation properties [56], the potentiodynamic additive transport behavior was further validated by EQCM measurement. Furthermore, electrochemical quartz crystal microbalance (EQCM) measurements were performed within a potential range prior to significant electrolyte reduction and subsequent SEI formation, where the observed mass change primarily reflects the accumulation of electrolyte species at the electrode interface (Figure S14). Consistent with the AIMD‐predicted solvation structures, AMS exhibits the strongest interaction with Li‐ions and is expected to be incorporated into the Li‐ion solvation structure to the greatest extent. Consequently, Li‐ions can transport a larger fraction of coordinated AMS molecules toward the negatively polarized electrode, leading to a greater increase in electrode mass. While AME and AMC depicted mass changes comparable to those of the background electrolyte, AMS exhibited a considerably larger mass increase with decreasing potential. This behavior indicates that Li‐ion‐associated AMS species are transported more effectively to the electrode interface, providing additional experimental support for the active participation of AMS in the Li‐ion solvation structure.

Figure 2 shows the initial interfacial characterization of the graphite electrode after formation in 1.0 A h graphite/LiNi_0.8_Co_0.1_Mn_0.1_O_2_ (NCM811) pouch cells with the injection of electrolytes to which AMS, AMC, and AME had been added, respectively. While a general additive evaluation was performed based on the weight percent of the additive in the electrolyte, a unified molar concentration–based electrolyte additive injection was performed to directly compare the SEI‐forming ability. The AMS, AMC, and AME additives (0.05 *M* concentration) were dissolved in the electrolyte to compare the SEI formation. During the formation cycle of Li‐ion batteries, the initial charging step involves the migration of Li ions from the positive electrode to the negative electrode. In this process, additives that exhibit a strong binding affinity for Li ions can spontaneously remain on the surface of the negative electrode. These additives, whose local concentration at the surface increases, are expected to promote the formation of a SEI film, thereby enabling the development of a more robust passivation layer. Based on this behavior, an analysis was conducted to evaluate whether the formation of an SEI layer on the graphite surface was effectively achieved. 3D maps obtained from time‐of‐flight secondary‐ion mass spectroscopy (ToF‐SIMS) were compared using the CH_3_O^−^ fractal, an SEI species typically derived from the allyl moiety (Figure 2a). The ToF‐SIMS maps clearly indicate that the intensified signal of the CH_3_O^−^ fractal increases with the Li‐ion binding energy of the additives. This indicates that the formation of a concentrated SEI film is possible through the participation of the additive in Li‐ion solvation. A comparison of the relative intensities of the C─O, O─C─O, and O─C═O peaks in the C 1s XPS spectra from the SEI film and the Li─C peak from the lithiated graphite can be used as a barometer of the thickness of the SEI films on the graphite electrode derived from the AMS, AMC, and AME molecules (Figure 2b). The C 1s spectra indicated that the initial SEI film was deposited in the sequence of AMS, AMC, and AME additives, further validating the initial SEI film characteristics. The allyl‐based SEI‐forming moiety has been reported to facilitate the reduction of EC molecules; hence, the bonding concentration of O─C═O and Li_2_CO_3_‐based species is high at the graphite surface with the AMS‐added electrolyte. Meanwhile, the concentration of the S‐based species in the SEI film was marginal (0.3 at.%) in the atomic concentration profile of the SEI film containing AMS (Figure S15). Figure 2c shows a schematic of the relationship between the Li‐ion binding ability and extent of SEI formation. The additive with the highest participation in Li ion solvation, AMS, was concentrated at the graphite surface as a result of spontaneous migration with Li ions during formation; therefore, effective formation of the initial SEI film on the graphite electrode is possible. In contrast, the additive with the lowest participation in Li ion solvation, AME, was randomly distributed throughout the electrolyte. Hence, the SEI film on the graphite electrode formed only with the limited amount of AME additive located near the graphite, thereby leading to an insufficiently thin initial SEI film. Consequently, the Li‐ion binding ability of the additive is one of the key determinants of the effective reinforcement of negative electrode–electrolyte interfaces.

**FIGURE 2:**
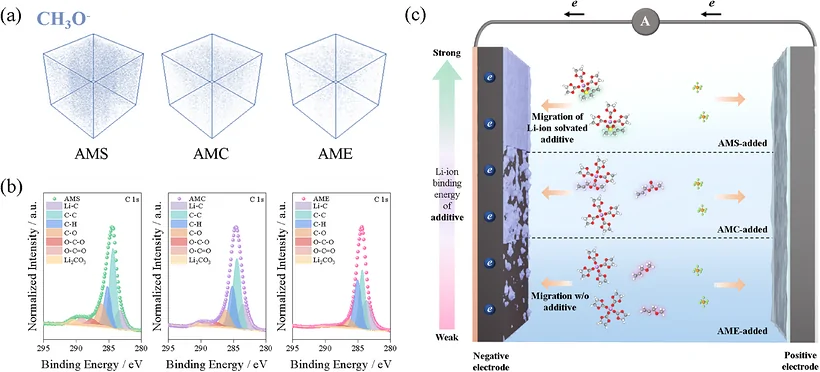
(a) 3D‐ToF‐SIMS maps; (b) C 1s XPS spectra of graphite electrodes with electrolytes containing AMS, AMC, and AME, respectively, as the additive after formation; (c) scheme of Li‐ion binding ability of additive toward effectiveness of SEI formation.

Figure 3 shows the relationship between the effectiveness of the initial SEI film formation and the cycling performance in practical pouch cells. The initial voltage profiles show that the 1.0 A h discharge capacity was similar for the three electrolytes (Figure S16). Assessment of the cycling performance at an elevated temperature (Figure 3a) revealed that the discharge capacity retention of cells with the additive‐containing electrolytes decreased in the order of AMS > AMC > AME, implying that the reinforcement and effective formation of the initial SEI film are significant for improving the cycling performance of the lithium‐ion battery system. Furthermore, the increase in the discharge‐end open‐circuit voltages (OCVs, Figure 3b) as cycling proceeded showed that the increasing tendency was suppressed by the AMS‐containing electrolyte. This indicated that the loss of the Li‐ion inventory in the cell and the discharge capacity loss resulting from the increase in the resistance were mitigated by the initially effective SEI reinforcement. To further validate practical visibility of the AMS additive, thousand‐cycle performance and pulse resistance measurements were performed with the background and AMS‐added electrolytes. The obtained capacity retention of the AMS‐added case was 71.1%, while the background electrolyte showed 64.1%, implying that interphasial reinforcement was effectively achieved by the AMS additive (Figure S17). Furthermore, the cycle‐number‐dependent DC‐iR resistance measurements demonstrate that the growth of internal resistance is significantly mitigated by AMS additive incorporation (Figure S18).

**FIGURE 3:**
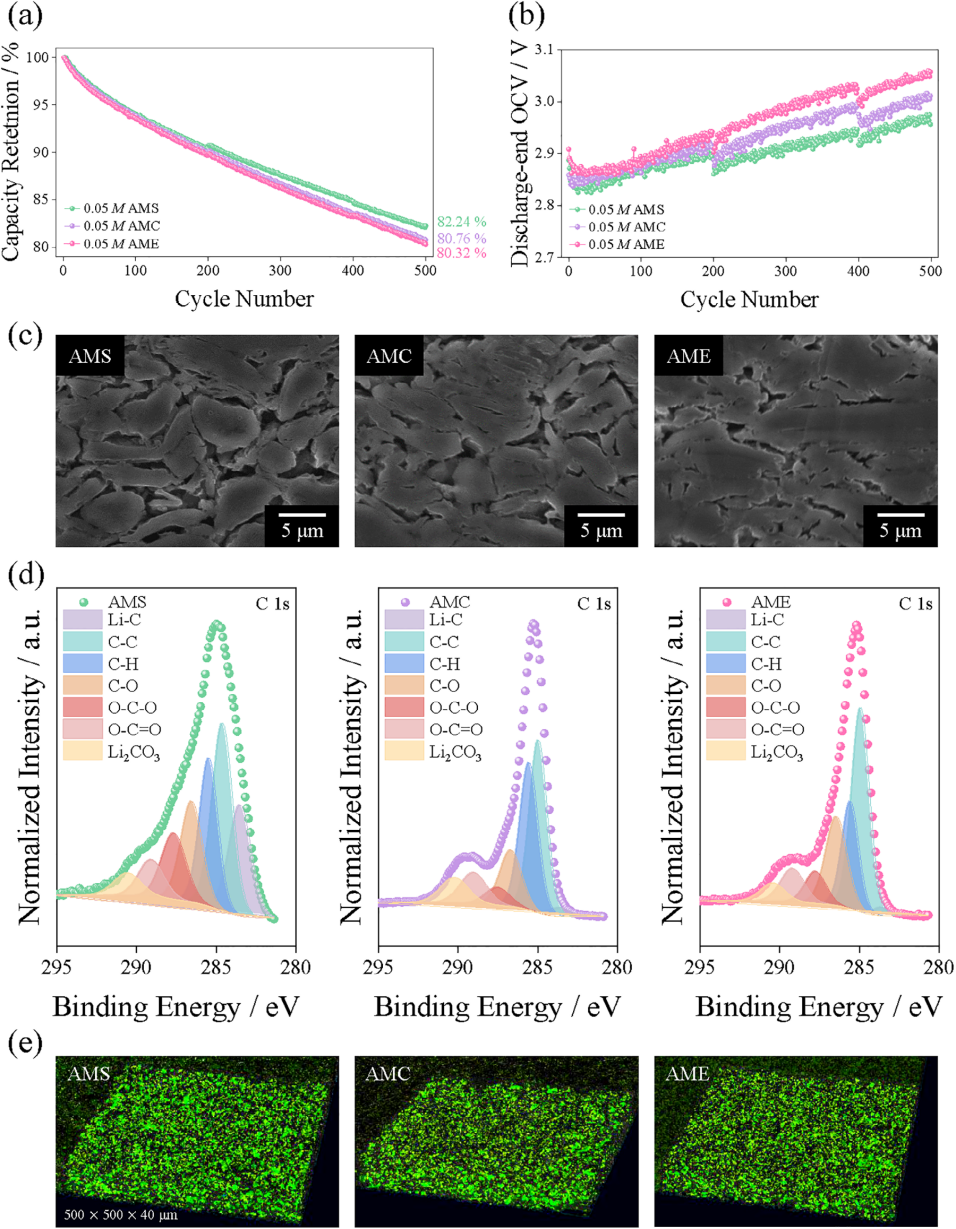
(a) 45°C, 1.0 C capacity retention, cycle number‐dependent (b) discharge‐end OCV performance; (c) cross‐sectional SEM images; (d) C 1s XPS spectra, (e) X‐ray microscopy maps with connected electrode pores (diameter > 1.5 µm) of cycled graphite electrodes in graphite/NCM811 1.0 A h cells with electrolytes containing AMS, AMC, and AME (0.05 M) as the additives.

Cross‐sectional scanning electron microscopy (SEM) images of the cycled graphite electrodes (Figure 3c) demonstrated that electrode pore clogging from the SEI film formed after the formation cycle is highly suppressed with the AMS‐containing electrolyte. In contrast, deposits of the SEI film are much more prominently visible for the AMC‐ and AME‐containing electrolytes (in this sequence) compared to those of the AMS‐containing electrolyte. This implies that the continued electrolyte decomposition on the graphite surface during cycling was suppressed by the SEI film reinforced by the AMS additive, predominantly as a result of its high Li‐ion binding energy. The C 1s XPS spectra (Figure 3d) also show that subsequent deposition of the SEI film is the highest at the graphite electrode with the AME‐containing electrolyte, as represented by the relative peak ratio between the Li─C and SEI species. The XPS analysis was performed to compare the relative intensities of SEI‐related signals such as C─O and C═O against the Li─C peak observed from the lithiated graphite electrode. Since the identical X‐ray source was used for XPS measurements, a relatively higher intensity of the Li─C signal in the C 1s spectra indicates less SEI film deposition, implying that the graphite signal is more prominently observed within the unified X‐ray penetration depth. Conversely, a lower Li─C signal intensity suggests more thick SEI formation and coverage on the electrode surface. In the C 1s spectra, AME exhibits a higher SEI‐related signal intensity compared to AMC and AMS, indicating more thick SEI film deposition after formation at AME. X‐ray microscopy (XRM) served to confirm that further pore structure deterioration induced by deposits from the SEI film was clearly visible on the graphite electrode (Figure 3e). The XRM maps indicate connected pores with diameters larger than 1.5 µm, which are typically observed pore sizes in the graphite electrode. Although intense green signals were observed for the graphite electrode with the AMS‐containing electrolyte, the intensity from the electrode pores decreased for the graphite electrodes with AMC‐ and AME‐containing electrolytes. Specifically, the porosities of the cycled graphite electrodes were 28.1, 24.0, and 19.5% for the AMS‐, AMC‐, and AME‐containing electrolytes, respectively (Figure S19). Hence, pore clogging of the negative electrode was highly suppressed by reinforcement of the initial SEI film, which was attributed to the increase in the concentration of the effective film‐forming additive in the formation step resulting from migration of the Li‐ion‐guided additive. The pore‐size distribution in the graphite electrode (Figure S20) also shows that the distribution of pores larger than 1.5 µm is the largest for AMS, followed by AMC and AME, the result of the formation of the SEI closer to the graphite electrode by the AMS additive. The Nyquist plots obtained from the Li/cycled graphite cells show that the growth of the SEI film and increase in the charge‐transfer resistance of the graphite electrode are largely mitigated in the presence of the AMS additive compared to the AMC and AME additives (Figure S21).

Figure 4 presents the *post‐mortem* analysis of the positive electrodes after cycling with the three different additives. Since these additives were specifically designed to reinforce the negative electrode interface, their impact on the positive electrode was expected to be minimal. Figure 4a shows the O 1s XPS spectra of the cycled positive electrodes. The similar intensities of the lattice oxygen signal across all three electrolytes indicate that the thickness of the deposited surface films on the positive electrodes remained similar. Furthermore, cross‐sectional SEM images in Figure 4b reveal that intergranular crack formation in the NCM811 particles was not evidently suppressed by any of the additives. These results confirm that the additive molecules primarily migrate toward the negative electrode along with the Li‐ion flux, meaning that positive electrode stabilization was not the dominant mechanism for the observed performance enhancement in the pouch cells.

**FIGURE 4:**
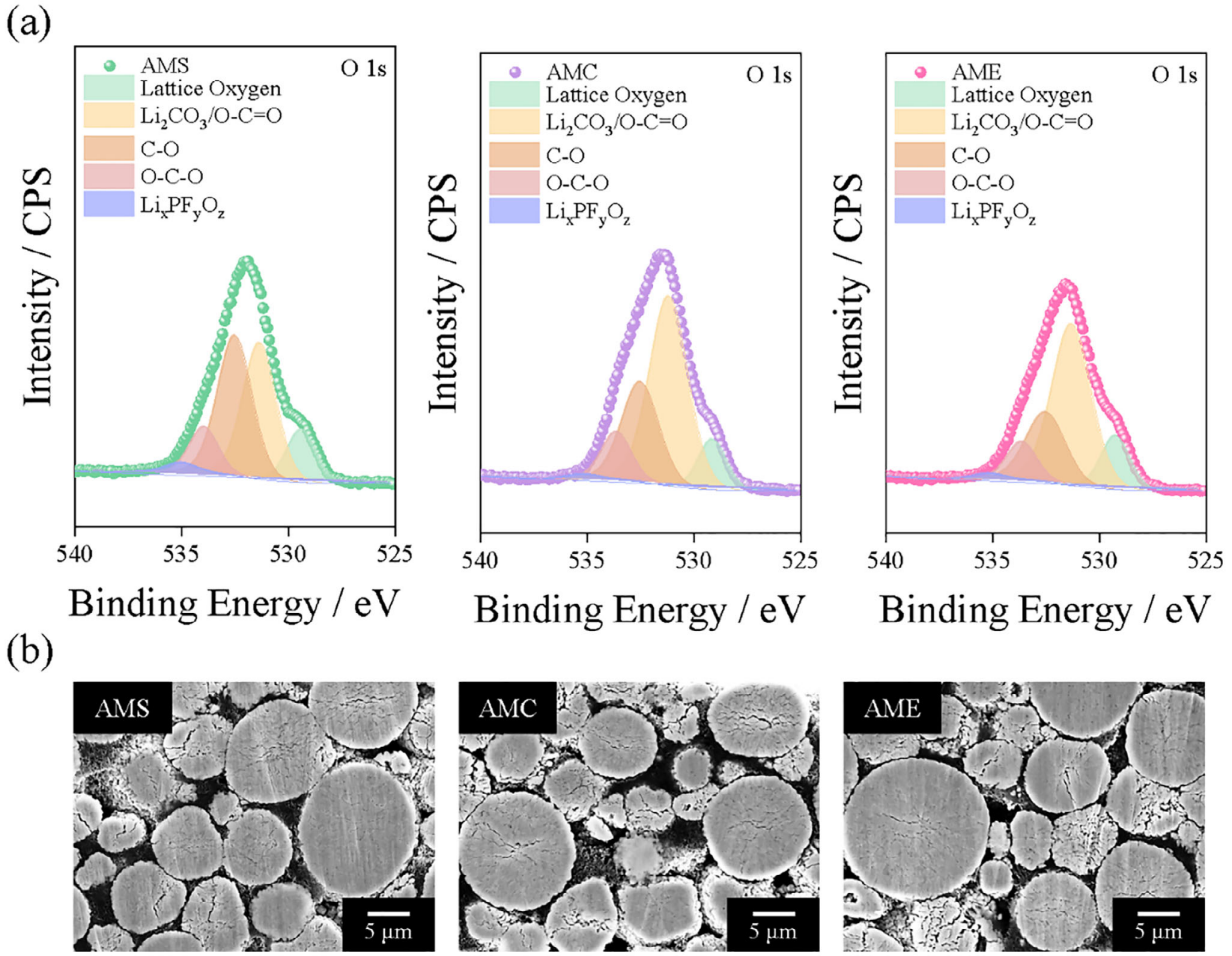
(a) XPS O 1s spectra and (b) cross‐sectional SEM images of NCM811 electrodes cycled in graphite/NCM811 cells with electrolyte containing AMS, AMC, and AME as the respective additives.

Figure 5 illustrates the proposed mechanism of solvation‐guided electrolyte additive design and its profound impact on the long‐term electrochemical stability of LIBs. The schematic shows that the Li‐ion binding affinity of an additive serves as an important determinant responsible for the effective reinforcement of the SEI. During the initial SEI formation process, additives with high Li‐ion binding energies, such as AMS, are preferentially incorporated into the Li‐ion solvation sheath. This allows the additive molecules to spontaneously migrate toward the surface of the negative electrode alongside the Li‐ion flux, with eventual localized enrichment of the film‐forming species at the electrode−electrolyte interface. This concentrated presence facilitates the construction of a robust and reinforced SEI layer that effectively passivates the graphite surface. In contrast, additives with weak binding affinities, such as AME, remain randomly distributed within the bulk electrolyte, resulting in a sparse initial passivation film that provides insufficient coverage of the electrode surface. As cycling progresses, the initially reinforced SEI derived from the high‐affinity additive successfully suppresses continuous electrolyte decomposition, thereby preventing pores within the electrode matrix from being clogged. The preservation of the porous structure ensures unimpeded Li‐ion transport and minimizes an increase in the resistance, whereas poorly passivated surfaces undergo sustained side reactions that clog the electrode pores, which causes rapid capacity decay. Ultimately, this solvation‐guided migration strategy provides a systematic pathway for designing additives that can selectively strengthen the electrode−electrolyte interface to improve long‐term performance.

**FIGURE 5:**
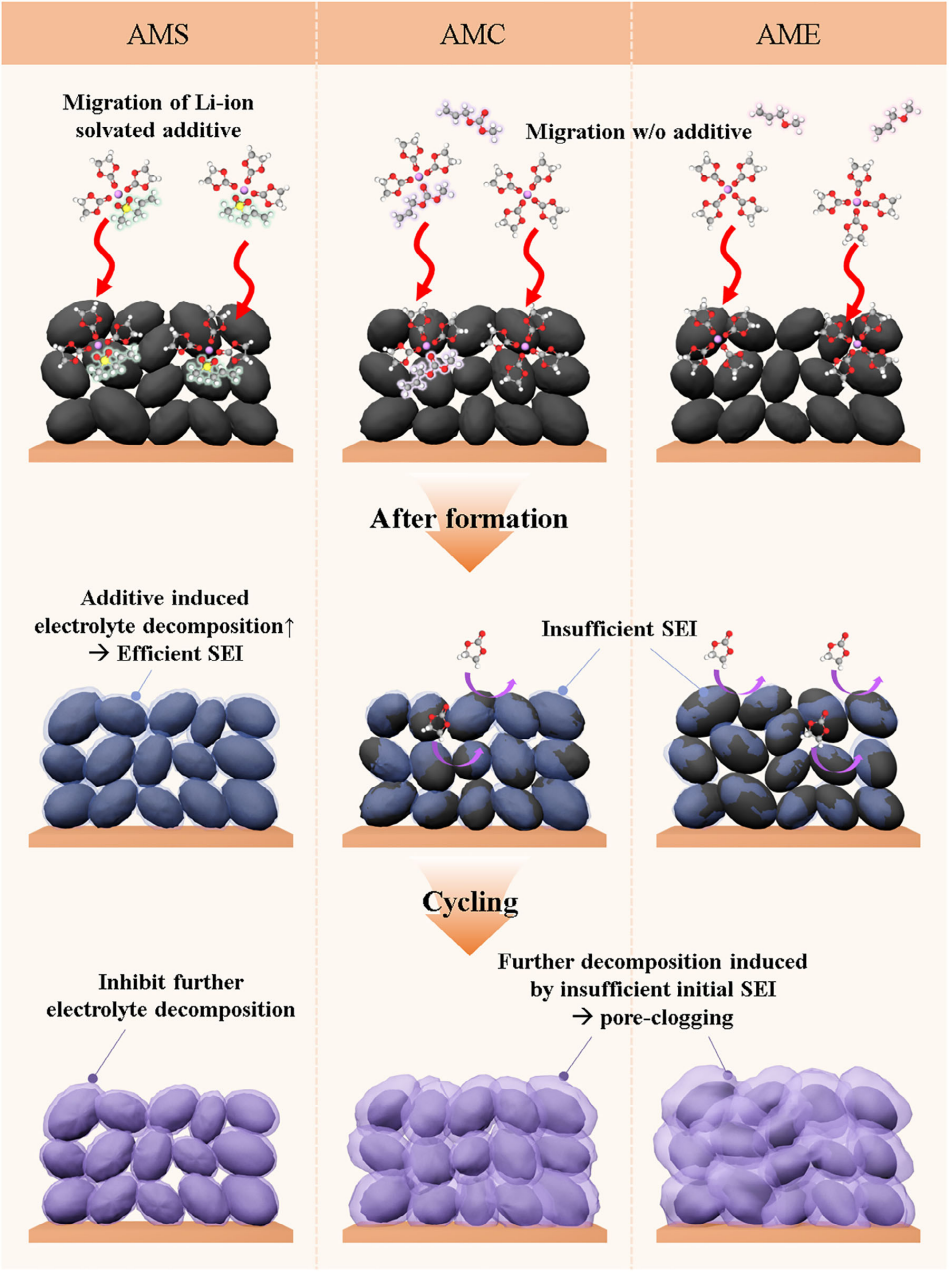
Illustration of the effectiveness of SEI formation guided by the ability of the additive to bind Li ions and the proposed mechanism of operation.

## 3 Conclusion

The Li‐ion binding energy of an electrolyte additive was shown to be the primary determinant of its ability to effectively form a robust SEI layer. By utilizing a system based on a σ‐bonding‐insulation model, we isolated the effect of solvation behavior from the thermodynamic reduction potential of the SEI‐forming moieties. Our results indicated that additives with strong Li‐ion binding affinities, such as AMS, are expected to exhibit a greater tendency toward transport to the graphite surface from solvation‐guided migration during the initial charging process. This localized enrichment enables the formation of a superior passivation layer that suppresses subsequent electrolyte side reactions. The practical significance of this design strategy was validated through 1.0 Ah pouch cell tests and X‐ray microscopy, which demonstrated that additives with a high binding affinity mitigate the pore‐clogging phenomenon and preserve the porous structure of the electrode over extended cycling. While SEI formation is governed by multiple factors, including reduction potentials and decomposition pathways, the present results demonstrate that Li‐ion binding energy provides an effective complementary descriptor for rational additive design. The proposed design strategy is expected to be particularly applicable to comparative evaluations of SEI‐forming additives with similar reactive moieties, where differences in decomposition chemistry are minimized. This study shifts the paradigm of additive design from a simple selection of functional atoms to a more sophisticated control of microsolvation structures and offers a powerful tool for the realization of long‐life and high‐energy‐density secondary batteries.

## Author Contributions

**Seoin Back**: Writing – review and editing, visualization, software, formal analysis. **Ho Yeon Jang**: methodology, software, visualization, formal analysis, writing – original draft. **Chihyun Hwang**: formal analysis, funding acquisition, supervision, resources. **Jooeun Byun**: methodology, data curation, investigation, formal analysis, validation, writing – original draft. **Chae Rim Lee**: formal analysis, methodology, validation. **Myung‐Jun Kwak**: methodology, visualization, formal analysis. **Hyun‐seung Kim**: conceptualization, software, data curation, investigation, validation, formal analysis, supervision, funding acquisition, visualization, project administration, resources, writing – original draft, writing – review and editing.

## Funding

This work was supported by the Ministry of Trade, Industry and Energy/Korea Evaluation Institute of Industrial Technology (MOTIE/KEIT) (Nos. RS‐2025‐25466171 and RS‐2025‐02432999). S.B. acknowledges the support from the National Research Foundation of Korea (NRF) grant funded by the Korea government (MSIT) (RS‐2025‐02214715) and generous supercomputing time from KISTI.

## Conflicts of Interest

The authors declare no conflicts of interest.

## Supporting information




**Supporting File**: advs77072‐sup‐0001‐SuppMat.docx

## Data Availability

The data that support the findings of this study are available from the corresponding author upon reasonable request.
