# Supplementary material for: Harnessing Li‐Ion Binding Energy for Solvation‐Guided Electrolyte Additive Design and Robust Solid Electrolyte Interphase Reinforcement
Source: Adv Sci (Weinh). 2026 Aug 10;13(61):e77072. doi: 10.1002/advs.77072 (PMC13455831; doi:10.1002/advs.77072)
Supplement: Supplementary file 1 [file ADVS-13-e77072-s001.docx]

Supporting Information

**Harnessing Li-ion Binding Energy for Solvation-Guided Electrolyte Additive Design and Robust Solid Electrolyte Interphase Reinforcement**

*Jooeun Byun, Ho Yeon Jang, Myung-Jun Kwak, Chae Rim Lee, Chihyun Hwang^,*^, Seoin Back^,*^, and Hyun-seung Kim^*^*

Experimental

***Electrolyte formulation and physicochemical characterization.*** Electrolyte formulation was conducted in an Ar-filled glove box. The AMS, AMC, and AME additives (Sigma-Aldrich) were exposed to activated molecular sieves (Sigma-Aldrich) for more than 12 h to remove moisture. The pre-dried additives were added to 1.0 *M* LiPF_6_ in ethylene carbonate (EC)/ethyl methyl carbonate (EMC) (3:7 = *v*/*v*; Dongwha Electrolyte) at a concentration of 0.05 *M* each. After the addition of the electrolyte, the mixture was stirred for 12 h to ensure complete dissolution. The solvation structures of the prepared electrolytes were analyzed using Raman spectroscopy (Thermo Fisher Scientific, DXR2xi). The ionic conductivities of the electrolytes were measured at 20 °C using a conductivity meter (Oakton CON 6+).

***Electrode and pouch cell fabrication.*** The positive electrode was prepared using a mixture of LiNi_0.8_Co_0.1_Mn_0.1_O_2_ (NCM811, BTR), conductive carbon (TIMCAL, Super P), and poly(polyvinylidene fluoride) (PVDF, Kureha, KF-1100) dispersed in *N*-methyl-2-pyrrolidone (NMP, Sigma-Aldrich) at a weight ratio of 96:2:2 (wt. %). The resulting slurry was cast on a 15 μm Al current collector and pre-dried at 80 °C before drying under vacuum overnight at 120 °C. The negative electrode was prepared by casting a deionized water-based slurry of artificial graphite (BTR), conductive carbon (TIMCAL, Super P), and styrene-butadiene rubber (SBR)/carboxymethyl cellulose (CMC) (1:1 wt.%, Sigma Aldrich) mixed in a ratio of 95:2.5:2.5 (wt.%). The slurry was cast on a 20 μm Cu current collector and pre-dried at 80 °C before drying under vacuum overnight at 80 °C. Pouch cells with a discharge capacity 1.2 A h and negative/positive electrode capacity ratio (N/P ratio) of 1.08 were fabricated by stacking the positive electrode, a polyethylene separator, and negative electrode. After injecting the additive-containing electrolyte (E/C ratio of 2.5), the stacked cells were rested at room temperature for 24 h. The cell was pressurized to 1.0 MPa using a jig to evaluate its electrochemical cycling performance.

***Electrochemical characterization of pouch cells.*** During film formation in the pouch cells (TOYO, Japan), a 0.1 *C* constant current (CC)–constant voltage (CV) charge (0.05 *C* cut-off) and a 0.1 *C* CC discharge were applied over a voltage range of 2.5–4.2 V at room temperature. After the formation process, cells were cycled with charging at 1.0 *C* CC–CV (0.05 C cut-off) and discharging at 1.0 *C* CC over the voltage range of 2.5–4.2 V at 45 °C.

***Electrochemical quartz crystal microbalance (EQCM) analysis.*** The sealed well-type EQCM cell was used with the Pt-sputtered quartz crystal electrode (QA-A9M, Seiko EG&G) in a dry room. The Pt-coated crystal was used as the working electrode, and Li metal (Honjo metal, 200 μm) was used as the counter and reference electrode. Mass changes of the electrode were evaluated in a background (1.0 *M* LiPF_6_ EC/EMC (3:7 = *v*/*v*)) and in 0.2 *M* additive-added electrolytes. To investigate interfacial mass deviation during low voltage exposure, a voltage was scanned from open-circuit voltage (OCV) to 0.3 V (*vs*. Li/Li^+^) at a scan rate of 5.0 mV s^−1^. The corresponding mass deviation was calculated based on the Sauerbrey equation with measured resonance frequency change.

***Linear sweep voltammetry (LSV).*** Measurements were performed using Li/Cu 2032 coin cells injected with 0.05 M additive containing electrolytes. The cells were assembled in dry-room. LSV experiments were conducted using a potentiostat instrument (BioLogics), and the voltage was swept from the OCV to 0 V at a scan rate of 0.1 mV s^-1^. All measurements were carried out under identical conditions to ensure reliable comparison of the electrochemical behavior.

***Electrochemical impedance spectroscopy*.** Electrochemical impedance spectroscopy (EIS) measurements were performed on Li/graphite half-cells reassembled in 2032 coin cells using electrodes dismantled from the graphite/NCM811 pouch cells after 500 cycles with the electrolyte 1.0 *M* LiPF_6_ EC/EMC (3:7 = *v*/*v*; Dongwha Electrolyte). After 12 h rest, measurements were performed at 50% state of charge (SOC) over the frequency range of 3.0 MHz to 10.0 mHz with 5.0 mV amplitude.

***Post-mortem spectroscopic analysis*.** Spectroscopic analyses of the electrodes were performed using XPS (Thermo Fisher Scientific), cross-sectional SEM (JEOL), and ToF-SIMS (ION-ToF) after SEI formation and cycling. For specimen preparation, the electrodes were rinsed with EMC (Soulbrain) to remove residual Li salt and solvent and dried under vacuum at room temperature for 12 h. To acquire cross-sectional SEM images of the electrode structures, the specimens were polished using a cryogenic cross-sectional polisher (cryo-CP, JEOL) at −50 °C.

All XPS spectra were calibrated by referencing the C–C peak in the C 1s spectrum to 284.4 eV.

***Computational Details.*** Electrostatic potential (ESP) maps of AMS, AMC, and AME were created using density functional theory (DFT) calculations using the Q-Chem 5.4.0 package ^[1]^. Geometric optimization was performed at the B3LYP/6-311++G(d,p) level **^[2]^** with a D3 (op) dispersion correction ^[3]^. The implicit solvation method using C-PCM **^[4]^** was employed with a dielectric constant of 18.5, representing LiPF_6_ in an EC:EMC (3:7) electrolyte **^[5]^**. All the structures were verified as true local minima in the absence of imaginary frequencies. Ab initio molecular dynamics (AIMD) simulations were conducted using the VASP 5.4.4 package **^[6]^**. We employed the projector augmented wave (PAW) method and the PBE functional, incorporating Grimme’s D3 correction for dispersion **^[7]^**. The calculations utilized a 500 eV kinetic energy cutoff, a Gamma-centered k-point mesh **^[8]^**, and convergence criteria of 10^-4^ eV for energy and 0.05 eV/Å for forces. Each AIMD system was composed of one Li^+^, one PF_6_^-^, six EC, 14 EMC, and one additive molecule (AMS, AMC, or AME). This composition yielded a salt concentration of 0.53 M. This dilute concentration (compared to the typical 1 M) was used to isolate the behavior of individual solvation structures by minimizing inter-ionic interactions ^[9]^. The free energy profiles (∆G) for the dissociation of each additive molecule (AMS, AMC, and AME) from the solvation structure were determined using the slow-growth approach implemented in VASP ^[10]^. These calculations started with pre-optimized structures, as depicted in **Figure S2** and **S3**. We defined the collective variable (CV), ξ, as the distance between the Li^+^ ion and the coordinating oxygen atom (O_bind_) of the additive. The CV was then elongated at a constant velocity of 10^-4^ Å/fs to map the detachment process. The potential of mean force (PMF) was obtained by integrating the constraint force along the CV path:

$$\Delta G=\int_{\xi_{1}}^{\xi_{2}} \left\langle f_{c} \right\rangle_{\xi} d\xi$$

Here, *<f_c_>_ξ_* represents the free energy gradient obtained from the Blue-moon ensemble ^[11]^, which is recorded as ‘*b_m*’ in the VASP ‘*REPORT*’ file.

Figure S1. The binding energies when a molecule occupies the (left) EMC coordination site and (right) EC coordination site. The molecular structures illustrate the corresponding optimized configurations, with the coordinating target molecule highlighted in yellow.

Figure S2. The binding energies of AMS, AMC and AME molecules within CIP structures

Figure S3. Lowest unoccupied molecular orbital energy levels of AMS, AMC and AME additives attached to Li ions, respectively.

**Figure S4.** Linear sweep voltammogram obtained from the 0.05 *M* additive-added electrolytes at Li/Cu cells

**Figure S5.** Illustration of the three pre-optimized additive systems. The [Li(EC)_2_(EMC)(additive)]^+^ complex was utilized as the initial solvation structure. During pre-optimization, the core Li-(O)_4_ cluster was constrained to stabilize the surrounding environment. The enlargements on the right illustrate the local solvation structures.

 Figure S6. Illustration of the three pre-optimized additive systems. The [Li(EC)(EMC)_2_(additive)]^+^ complex was utilized as the initial solvation structure. During pre-optimization, the core Li-(O)_4_ cluster was constrained to stabilize the surrounding environment. The enlargements on the right illustrate the local solvation structures.

 **Figure S7.** Snapshots of the initial and final states, along with energy profiles, for the 30 ps AIMD simulations of each additive system (AMS, AMC and AME). The [Li(EC)_2_(EMC)(additive)]^+^ complex was utilized as the initial solvation structure. The energy distributions were calculated from the production trajectory (10–30 ps).

 Figure S8. Snapshots of the initial and final states, along with the energy profiles, for the 30 ps AIMD simulations of each additive system (AMS, AMC, and AME). The [Li(EC)(EMC)_2_(additive)]^+^ complex was utilized as the initial solvation structure. The energy distributions were calculated from the production trajectory (10–30 ps).

***Supplementary note 1*: Slow-growth AIMD simulations for the [Li(EC)(EMC)_2_(additive)]^+^ solvation structure.**

We additionally investigated the detachment process in the [Li(EC)(EMC)_2_(additive)]^+^ solvation environment using slow-growth AIMD (**Figure S9** and **S10**). Initially, the Li-O_bind_ distance was employed as the CV for all the additive systems. However, in the AMS system, extending the Li-O_bind_ CV beyond 1.0 Å led to an artifact where an adjacent oxygen atom from the sulfonate group re-coordinated with the Li^+^. To enforce the complete detachment of the AMS, we modified the simulation pathway by switching the CV from the Li-O_bind_ distance to the distance between the Li^+^ and the sulfur atom of AMS (Li-S_AMS_) after reaching a 1.0 Å displacement of the Li-O_bind_ bond. As depicted by the green dashed line in **Figure S10**, this adjusted CV successfully induced full detachment of AMS to yield a continuously increasing free energy profile. Detachment of the AMC and AME systems was successfully achieved using the original Li-O_bind_ CV system without re-coordination.

 Figure S9. Snapshots of the slow-growth simulations for the [Li(EC)(EMC)_2_(additive)]^+^ systems. The structures were captured at specific collective variable (CV) displacements: 0.0 Å (0 ps), 1.0 Å (10 ps), and 2.0 Å (20 ps). For the AMS system, the snapshots demonstrate the effect of switching the CV definition after reaching a 1.0 Å displacement of the Li-O_bind_ distance.

Figure S10. Free energy profiles (∆G) for the detachment of AMS, AMC, and AME from the initial [Li(EC)(EMC)_2_(additive)]^+^ solvation structures, calculated using slow-growth AI-MD. For the AMC and AME, the profiles were obtained using the standard collective variable (CV), defined as the Li-O_bind_ distance, throughout the entire process. For the AMS, to ensure complete detachment without re-coordination with Li^+^ ion, the CV was switched from the Li-O_bind_ distance to the Li-S_AMS_ distance after an initial 1.0 Å displacement. The shaded region (CV > 1.0 Å) indicates the phase where this adjusted CV was applied for the AMS.

**Figure S11**. The reductive energies for AMS, AMC, and AME. The calculations compare the isolated single molecules (left) with the Li+ solvation complexes where the additive occupies either an EMC (middle) or EC (right) site.

**Figure S12.** Concentration profile of Li-ion-attached and detached species of AMS, AMC, and AME molecules.

**Figure S13.** Ionic conductivity of electrolytes containing 1.0 wt.% and 3.0 wt.% of the additives AMS, AMC, and AME.

**Figure S14.** voltage *vs.* electrode mass change profiles obtained from the EQCM measurement.

**Figure S15.** Atomic concentration profile of the AMS-added SEI film.

**Figure S16.** Initial voltage profiles of pouch cells at 45 °C. The cells were injected with electrolyte to which AMS, AMC, and AME, respectively, had been added.

**Figure S17.** 45 ℃, 1.0 *C* 1000 cycle capacity retention with background and 0.05 *M* AMS-added electrolytes.

**Figure S18.** cycle number-dependent DC-*iR* with background and additive-added electrolytes.

**Figure S19.** Porosity of the cycled graphite electrodes

**Figure S20.** Pore-size distribution of the cycled graphite electrode

**Figure S21.** Nyquist plots of the reassembled Li/graphite cells obtained from the cycled graphite electrode with electrolyte to which AMS, AMC, and AME had been added.

**References**

[1]	E. Epifanovsky, A. T. B. Gilbert, X. Feng, J. Lee, Y. Mao, N. Mardirossian, P. Pokhilko, A. F. White, M. P. Coons, A. L. Dempwolff, Z. Gan, D. Hait, P. R. Horn, L. D. Jacobson, I. Kaliman, J. Kussmann, A. W. Lange, K. U. Lao, D. S. Levine, J. Liu, S. C. McKenzie, A. F. Morrison, K. D. Nanda, F. Plasser, D. R. Rehn, M. L. Vidal, Z.-Q. You, Y. Zhu, B. Alam, B. J. Albrecht, A. Aldossary, E. Alguire, J. H. Andersen, V. Athavale, D. Barton, K. Begam, A. Behn, N. Bellonzi, Y. A. Bernard, E. J. Berquist, H. G. A. Burton, A. Carreras, K. Carter-Fenk, R. Chakraborty, A. D. Chien, K. D. Closser, V. Cofer-Shabica, S. Dasgupta, M. de Wergifosse, J. Deng, M. Diedenhofen, H. Do, S. Ehlert, P.-T. Fang, S. Fatehi, Q. Feng, T. Friedhoff, J. Gayvert, Q. Ge, G. Gidofalvi, M. Goldey, J. Gomes, C. E. González-Espinoza, S. Gulania, A. O. Gunina, M. W. D. Hanson-Heine, P. H. P. Harbach, A. Hauser, M. F. Herbst, M. Hernández Vera, M. Hodecker, Z. C. Holden, S. Houck, X. Huang, K. Hui, B. C. Huynh, M. Ivanov, Á. Jász, H. Ji, H. Jiang, B. Kaduk, S. Kähler, K. Khistyaev, J. Kim, G. Kis, P. Klunzinger, Z. Koczor-Benda, J. H. Koh, D. Kosenkov, L. Koulias, T. Kowalczyk, C. M. Krauter, K. Kue, A. Kunitsa, T. Kus, I. Ladjánszki, A. Landau, K. V. Lawler, D. Lefrancois, S. Lehtola, R. R. Li, Y.-P. Li, J. Liang, M. Liebenthal, H.-H. Lin, Y.-S. Lin, F. Liu, K.-Y. Liu, M. Loipersberger, A. Luenser, A. Manjanath, P. Manohar, E. Mansoor, S. F. Manzer, S.-P. Mao, A. V. Marenich, T. Markovich, S. Mason, S. A. Maurer, P. F. McLaughlin, M. F. S. J. Menger, J.-M. Mewes, S. A. Mewes, P. Morgante, J. W. Mullinax, K. J. Oosterbaan, G. Paran, A. C. Paul, S. K. Paul, F. Pavošević, Z. Pei, S. Prager, E. I. Proynov, Á. Rák, E. Ramos-Cordoba, B. Rana, A. E. Rask, A. Rettig, R. M. Richard, F. Rob, E. Rossomme, T. Scheele, M. Scheurer, M. Schneider, N. Sergueev, S. M. Sharada, W. Skomorowski, D. W. Small, C. J. Stein, Y.-C. Su, E. J. Sundstrom, Z. Tao, J. Thirman, G. J. Tornai, T. Tsuchimochi, N. M. Tubman, S. P. Veccham, O. Vydrov, J. Wenzel, J. Witte, A. Yamada, K. Yao, S. Yeganeh, S. R. Yost, A. Zech, I. Y. Zhang, X. Zhang, Y. Zhang, D. Zuev, A. Aspuru-Guzik, A. T. Bell, N. A. Besley, K. B. Bravaya, B. R. Brooks, D. Casanova, J.-D. Chai, S. Coriani, C. J. Cramer, G. Cserey, A. E. DePrince, III, R. A. DiStasio, Jr., A. Dreuw, B. D. Dunietz, T. R. Furlani, W. A. Goddard, III, S. Hammes-Schiffer, T. Head-Gordon, W. J. Hehre, C.-P. Hsu, T.-C. Jagau, Y. Jung, A. Klamt, J. Kong, D. S. Lambrecht, W. Liang, N. J. Mayhall, C. W. McCurdy, J. B. Neaton, C. Ochsenfeld, J. A. Parkhill, R. Peverati, V. A. Rassolov, Y. Shao, L. V. Slipchenko, T. Stauch, R. P. Steele, J. E. Subotnik, A. J. W. Thom, A. Tkatchenko, D. G. Truhlar, T. Van Voorhis, T. A. Wesolowski, K. B. Whaley, H. L. Woodcock, III, P. M. Zimmerman, S. Faraji, P. M. W. Gill, M. Head-Gordon, J. M. Herbert, A. I. Krylov, *The Journal of Chemical Physics* **2021**, 155.

[2]	a) C. Lee, W. Yang, R. G. Parr, *Physical Review B* **1988**, 37, 785; b) S. F. Boys, F. Bernardi, *Mol. Phys.* **1970**, 19, 553.

[3]	J. Witte, N. Mardirossian, J. B. Neaton, M. Head-Gordon, *J. Chem. Theory Comput.* **2017**, 13, 2043.

[4]	V. Barone, M. Cossi, *The Journal of Physical Chemistry A* **1998**, 102, 1995.

[5]	J. Self, N. T. Hahn, K. A. Persson, *ENERGY & ENVIRONMENTAL MATERIALS* **2024**, 7, e12494.

[6]	G. Kresse, J. Furthmüller, *Computational Materials Science* **1996**, 6, 15.

[7]	a) S. Grimme, J. Antony, S. Ehrlich, H. Krieg, *The Journal of Chemical Physics* **2010**, 132, 154104; b) G. Kresse, D. Joubert, *Physical Review B* **1999**, 59, 1758; c) P. E. Blöchl, *Physical Review B* **1994**, 50, 17953.

[8]	S. Froyen, *Physical Review B* **1989**, 39, 3168.

[9]	a) M. T. Ong, O. Verners, E. W. Draeger, A. C. T. van Duin, V. Lordi, J. E. Pask, *The Journal of Physical Chemistry B* **2015**, 119, 1535; b) M. A. Lee, H. Y. Jang, J. Lee, J. Byun, Y. Jung, J. H. Song, J. S. Yu, H. K. Song, C. Hwang, S. Back, H. S. Kim, *Small* **2025**, 21, e2407910.

[10]	a) T. K. Woo, P. M. Margl, P. E. Blöchl, T. Ziegler, *The Journal of Physical Chemistry B* **1997**, 101, 7877; b) C. Jarzynski, *Phys. Rev. Lett.* **1997**, 78, 2690.

[11]	a) W. K. Den Otter, W. J. Briels, *Mol. Phys.* **2000**, 98, 773; b) E. A. Carter, G. Ciccotti, J. T. Hynes, R. Kapral, *Chem. Phys. Lett.* **1989**, 156, 472.
